# Translating and Validating the Japanese Version of the Tolerance for Ambiguity Scale

**DOI:** 10.1007/s40670-024-02269-5

**Published:** 2024-12-30

**Authors:** Hirohisa Fujikawa, Takayuki Ando, Kayo Kondo, Mikio Hayashi

**Affiliations:** 1 Center for General Medicine Education, School of Medicine, Keio University, 35 Shinanomachi, Shinjuku-Ku, Tokyo, 160-8582 Japan; 2 Department of Medical Education Studies, International Research Center for Medical Education, Graduate School of Medicine, The University of Tokyo, Bunkyo-Ku, Tokyo, Japan; 3 School of Modern Languages and Cultures, Durham University, Durham, UK; 4 Center for Health Professions Education, Kansai Medical University, Hirakata, Osaka Japan

**Keywords:** Tolerance for ambiguity, Medical students, Japan, Factor analysis

## Abstract

**Background:**

Ambiguity is inherent in medicine, and tolerance for ambiguity (TFA) has recently been of substantial interest. Effective medical education for TFA requires a validated inventory; one validated measure in wide use is the seven-item TFA scale. In Japan, however, a tool for measuring TFA in undergraduate medical education has not been available. Here, we aimed to develop and validate the Japanese version of the TFA scale (J-TFA scale).

**Methods:**

We translated the original English scale into Japanese following an international guideline. We then conducted a validation survey by distributing an online anonymous self-administered questionnaire to medical students at three medical schools in Japan. We assessed the structural validity and internal reliability of consistency of the scale.

**Results:**

A total of 399 participants were included in our analysis. We used a split-half validation approach, with exploratory factor analysis (EFA) on the first half and confirmatory factor analysis (CFA) on the second. EFA indicated a two-factor structure. CFA showed that the two-factor structure suggested by EFA had acceptable model fitness indices. Cronbach’s alpha was 0.72, exceeding the satisfactory internal reliability consistency criteria.

**Conclusions:**

The J-TFA scale was developed and its psychometric properties were confirmed. This instrument may be useful for future educational interventions and research on TFA.

## Background

Ambiguity is inherent and inevitable in the practice of medicine. Ambiguity is defined as the absence of reliable, credible, or adequate information [1]. Daily clinical practice is full of ambiguity, including limitations of medical knowledge, ambiguous diagnostic problems, and ambiguities of therapy and outcome [2]. Despite significant advances in medical knowledge and technology, ambiguity is not disappearing. In fact, ambiguities appear to be increasing, consistent with the growing number of patients with multimorbidity, patient diversity, and social responsibility in medical education. These factors highlight the need for more inclusive educational and clinical approaches [3, 4].

The ability to cope with ambiguity is therefore a critical characteristic of physicians, and tolerance for ambiguity (TFA) is of considerable interest. TFA refers to how one perceives, responds to, and tolerates a situation where information is potentially unreliable, not credible, or inadequate [1, 5, 6]. The TFA of physicians affects patient outcomes and is associated with the mental health and well-being of physicians [7–10]. Previous studies have indicated relationships between TFA and patient care; among these, lower physician TFA may lead to more negative attitudes toward underserved populations [11], increased ordering of diagnostic tests [12], and greater fear of making mistakes [13]. In addition, higher TFA may be associated with greater leadership abilities and more empathic patient care [14, 15]. Given the importance of nurturing TFA among physicians and medical trainees, ensuring effective education about TFA requires a validated assessment scale.

Among the several scales for measuring the TFA of medical trainees or physicians currently available, the TFA scale is one of the most widely studied measures and has received attention in the field of medical education [16]. Geller et al. developed a modified scale for measuring TFA in 1993. The scale has several advantages. First, its reliability and validity have been comprehensively evaluated [16]. Second, it is relatively short and easy to administer. Shorter questionnaires can decrease the response burden, which can in turn lead to higher response rates, higher completion, and higher data quality [17]. Third, the scale can be applied to preclinical medical students because it measures general ambiguity tolerance that is not specific to the clinical context. Accordingly, it has been widely used in Western countries. In particular, in the USA, the scale was included in the Association of American Medical Colleges Matriculating Student Questionnaire in 2013 [18], and since then, more than 10,000 matriculating US medical students have completed it each year.

Conversely, in Japan, activities for assessing the TFA of medical trainees and physicians have just begun in limited settings, and undergraduate medical education focused on ambiguity tolerance is insufficient. In fact, Spector et al. indicated that Japan has one of the lowest levels of ambiguity tolerance among the 23 countries surveyed in cross-cultural and cross-national research [19], and it is assumed that this is the case for medical trainees in Japan. However, to our knowledge, no measures are available for assessing the TFA of preclinical medical students in Japan. Accordingly, developing and validating a Japanese version of the TFA scale (J-TFA scale) is of high priority.

Here, we aimed to translate the TFA scale into Japanese and examine its psychometric properties. Our scale will be helpful in informing the design and assessing the effectiveness of educational interventions aimed at nurturing TFA in Japan. The development of the scale could also lead to further international research on ambiguity tolerance.

## Methods

### Design, Setting, and Participants

This multicenter, cross-sectional study was performed in May 2024. The study formed a part of a research project regarding the medical professionalism of medical students. Medical students at three medical schools were invited to participate in our study through the directors of medical education of each of the medical schools. We informed them of the voluntary and anonymous nature of participation and that there were no disadvantages for not participating. We asked the participants to check the consent box at the beginning of the questionnaire to indicate their willingness to participate in the study, and only those who agreed to participate were enrolled.

Participants were asked to complete an online self-administered questionnaire using SurveyMonkey (www.surveymonkey.com). Non-respondents were reminded several times via e-mail to complete the survey. As an incentive, a gift card worth 3000 yen was given to ten winners drawn by lottery.

### Measures

#### Original English Scale by Gelleret al*.*

The original TFA scale by Geller et al. has seven items [16]. The items are rated on a 6-point Likert scale (1 = strongly agree to 6 = strongly disagree). Factor analysis showed that the scale has two subscales: “willingness to admit discomfort with ambiguity” (Q1–3) and “desire for certainty” (Q4–7). The scores of the scale are calculated by simply summing the responses to all seven items, giving a range of 7 to 42, with higher scores corresponding to greater tolerance of ambiguity.

#### Procedure for Translation

At our request, the original author of the TFA scale readily agreed to our translation of the scale into Japanese and development of a Japanese version. In accordance with the cross-cultural adaptation guideline suggested by Beaton et al. [20], we translated the original English scale into Japanese through the following steps. First, forward translation was carried out independently by the three authors (HF, TA, and KK), each of whom spoke Japanese natively. HF and TA had research experience regarding ambiguity tolerance [8, 15] and are familiar with the concept, while HF and KK have extensive experience in translating questionnaires in the field of medical education [21–23]. Second, the three translators checked the results of the forward translations. Discrepancies in the forward translations were resolved by repeated discussions, leading to the development of a reconciled version of the translations (Ver. 1). Third, the reconciled version (Ver. 1) was translated back into English by a professional bilingual translator who was not involved in our study. HF, TA, and KK compared the back-translated version with the original English version and proofread it (Ver. 2). Fourth, the three authors asked a medical education expert (MH) to review the proofread version (Ver. 2). We received feedback from the expert and further revised it accordingly (Ver. 3). Fifth, we asked the original author of the English scale to review Ver. 3, and thereafter modified it (Ver. 4). Finally, we conducted a pilot test with three medical trainees and cognitive debriefing, which showed no problematic items. Thus, we concluded that Ver. 4 was the final version.

### Statistical Analysis

Exploratory factor analysis and confirmatory factor analysis (EFA and CFA, respectively) were conducted to assess the structural validity of the J-TFA scale. Given the issues with performing both EFA and CFA on the same sample [24], we randomly divided the study participants into two groups, one half for EFA and the second half for CFA. While our initial hypothesis was that the Japanese version of the scale would have a similar two-factor structure to the English version, we recognized that the distinctive Japanese context may influence the factor structure. Since we were required to develop a scale that is suitable for the Japanese context, we decided to perform the EFA first.

Before analysis, the suitability of the data set for EFA was verified using the Kaiser–Meyer–Olkin (KMO) and Bartlett tests. The KMO value should exceed 0.60 and Bartlett’s test of sphericity should be significant [25]. We then performed EFA using the maximum likelihood with promax rotation method. We determined the number of factors (i.e., hidden or underlying variables inferred from a set of directly measurable variables) with reference to the results of the parallel analysis [26]. Factor loadings are acceptable at 0.30 or above, practically significant at 0.50 or above, and indicative of a well-defined structure at 0.70 or above [26]. Accordingly, we used a threshold of 0.30 for acceptable factor loadings.

Subsequently, CFA was performed using the maximum likelihood estimation approaches. We calculated the following model fitness indices: comparative fit index (CFI), root mean square error of approximation (RMSEA), and standardized root mean square residual (SRMR). CFI is considered acceptable at 0.90 or greater. RMSEA is characterized as follows: < 0.08, good; 0.08–0.10, moderate. SRMR is considered to be acceptable at 0.08 or below [27].

The Cronbach’s alpha was used to assess the internal consistency reliability of the J-TFA scale. The value of Cronbach’s alpha was computed using data of the entire sample (*n* = 399, as will hereinafter be described in detail). The values are characterized as follows: < 0.50, insufficient; 0.50 to 0.69, moderate; 0.70 to 0.79, satisfactory; and ≥ 0.80, good [28]. Finally, we demonstrated descriptive statistics of the scale. For all statistical analysis, we chose complete case analysis. Our data were analyzed using R version 4.4.0.

### Ethical Considerations

This study obtained ethical clearance from the ethics committee of Keio University School of Medicine (20231223).

## Results

Of the total 2170 eligible participants, 399 (18.4%) completed the survey. There were no missing data. Table 1 shows the profile of the participants; 251 (62.9%) were male. Most of the participants were in their 4th and 1st year (129 (32.3%) and 93 (23.3%), respectively). Table 2 presents the participants’ responses to the J-TFA scale.

**Table 1:** Participants’ characteristics (*N* = 399)

Characteristics	*n* (%)
Gender	
Female	146 (36.6)
Male	251 (62.9)
Others	2 (0.5)
Year	
1st	93 (23.3)
2nd	35 (8.8)
3rd	50 (12.5)
4th	129 (32.3)
5th	54 (13.5)
6th	38 (9.5)

**Table 2:** Responses to the questionnaire (*N* = 399)

Items	Responses, *n* (%)					
	1 = Strongly Agree	2 = Moderately Agree	3 = Slightly Agree	4 = Slightly Disagree	5 = Moderately Disagree	6 = Strongly Disagree
Q1^a^	16 (4.0)	116 (29.1)	133 (33.3)	64 (16.0)	57 (14.3)	13 (3.3)
Q2^b^	62 (15.5)	152 (38.1)	104 (26.1)	42 (10.5)	27 (6.8)	12 (3.0)
Q3^c^	25 (6.3)	68 (17.0)	104 (26.1)	65 (16.3)	107 (26.8)	30 (7.5)
Q4^d^	95 (23.8)	137 (34.3)	99 (24.8)	32 (8.0)	25 (6.3)	11 (2.8)
Q5^e^	22 (5.5)	61 (15.3)	87 (21.8)	89 (22.3)	105 (26.3)	35 (8.8)
Q6^f^	35 (8.8)	56 (14.0)	73 (18.3)	63 (15.8)	107 (26.8)	65 (16.3)
Q7^g^	39 (9.8)	89 (22.3)	94 (23.6)	84 (21.1)	66 (16.5)	27 (6.8)

Original English items were as follows:

^a^It really disturbs me when I am unable to follow another person’s train of thought

^b^If I am uncertain about the responsibilities involved in a particular task, I get very anxious

^c^I am often uncomfortable with people unless I feel that I can understand their behavior

^d^Before any important task, I must know how long it will take

^e^I don’t like to work on a problem unless there is a possibility of getting a clear-cut and unambiguous answer

^f^The best part of working on a jigsaw puzzle is putting in that last piece

^g^A good task is one in which what is to be done and how it is to be done are always clear

### Structural Validity Analysis

**Fig. 1:**
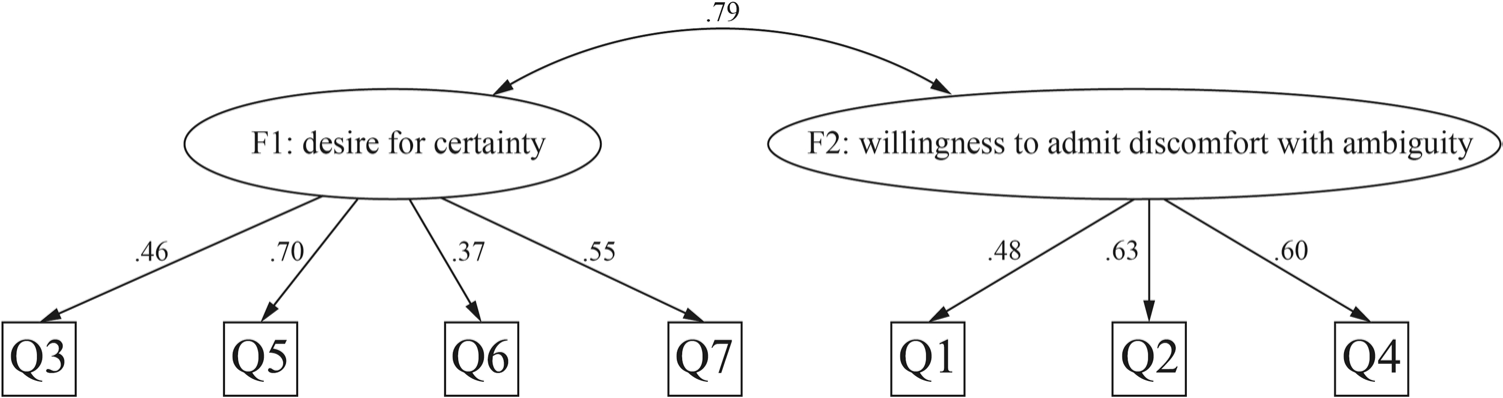
Factor structure of the Japanese version of the tolerance for ambiguity scale (confirmatory factor analysis). Ellipses are latent variables (factors). Squares are observed variables (items). Values on single-headed arrows are standardized factor loadings. Values on double-headed arrows are correlation coefficients; F, factors; Q, questions

As the value of KMO was 0.75 and Bartlett sphericity test was significant (*p* < 0.05), we decided to carry out EFA on the half sample (*n* = 186). The parallel analysis suggested a two-factor solution. Then, factor extraction was conducted. Table 3 shows the final results of the EFA. We then conducted CFA on the second sample (*n* = 213) (Fig. 1), which indicated that the model fitness of the J-TFA scale with the two-factor structure suggested by the EFA result was acceptable (CFI 0.909, RMSEA 0.083, SRMR 0.051). With reference to the original English scale, we named Factor 1 as “desire for certainty” and Factor 2 as “willingness to admit discomfort with ambiguity.”

**Table 3:** Results of the exploratory factor analysis (*N* = 186)

Items (as in original English version)	Factor loadings	
	1	2
Q1^a^	0.24	**0.42**
Q2^b^	− 0.24	**0.99**
Q3^c^	**0.46**	0.08
Q4^d^	0.23	**0.39**
Q5^e^	**0.90**	− 0.23
Q6^f^	**0.31**	0.07
Q7^g^	**0.62**	0.01
	**Values**	
Eigenvalue	2.12	0.31
Percentage variance explained	22	18

Original English items were as follows:

^a^It really disturbs me when I am unable to follow another person’s train of thought

^b^If I am uncertain about the responsibilities involved in a particular task, I get very anxious

^c^I am often uncomfortable with people unless I feel that I can understand their behavior

^d^Before any important task, I must know how long it will take

^e^I don’t like to work on a problem unless there is a possibility of getting a clear-cut and unambiguous answer

^f^The best part of working on a jigsaw puzzle is putting in that last piece

^g^A good task is one in which what is to be done and how it is to be done are always clear

### Internal Consistency Reliability Analysis and Descriptive Statistics

The value of the Cronbach’s alpha for all 7 items were 0.72 (satisfactory). The Cronbach’s alpha values for Factors 1 and 2 were 0.60 (moderate) and 0.62 (moderate), respectively. Descriptive statistics are shown in Table 4. We also show descriptive data by subgroup (Table 5). Through this process, we successfully developed the final version of the J-TFA scale (Table 6).

**Table 4:** Internal consistency reliability analysis and descriptive statistics

	Number of items	Mean	Standard deviation	Observed range	Cronbach’s alpha
Total	7	22.9	5.8	7–42	0.72
Factor 1	4	14.6	3.9	4–24	0.60
Factor 2	3	8.3	2.8	3–18	0.62

**Table 5:** Descriptive data by subgroup

	Total^a^, mean (SD)	Factor 1^b^, mean (SD)	Factor 2^c^, mean (SD)
Gender			
Female	22.4 (5.0)	14.6 (3.7)	7.8 (2.4)
Male	23.1 (6.2)	14.5 (4.0)	8.6 (3.0)
Others	24.5 (0.7)	15.5 (0.7)	9.0 (1.4)
Year			
1	22.7 (5.4)	14.9 (4.0)	7.8 (2.6)
2	21.8 (5.5)	13.4 (3.3)	8.3 (3.1)
3	21.6 (5.4)	13.7 (3.8)	7.9 (2.6)
4	23.7 (6.3)	14.8 (4.0)	8.9 (3.0)
5	23.4 (5.3)	15.3 (3.6)	8.1 (2.6)
6	22.2 (6.1)	14.2 (4.2)	8.0 (2.6)

*SD* standard deviation

^a^Ranging from 7 to 42, with higher scores indicating greater tolerance for ambiguity

^b^Ranging from 4 to 24, with higher scores indicating greater tolerance for ambiguity

^c^Ranging from 3 to 18, with higher scores indicating greater tolerance for ambiguity

**Table 6:** The Japanese version of the tolerance for ambiguity scale

No	English version	Japanese version
1	It really disturbs me when I am unable to follow another person’s train of thought	私は、他人の思考回路についていけないと、本当に困惑する。
2	If I am uncertain about the responsibilities involved in a particular task, I get very anxious	私は、特定の仕事に関わる責任が不確かだと、非常に不安になる。
3	I am often uncomfortable with people unless I feel that I can understand their behavior	私は、人の行動が理解できると思えない限り、その人に対してよく不愉快な気持ちになる。
4	Before any important task, I must know how long it will take	私は、重要な仕事の前には、それにどのぐらいの時間がかかるかを知っていなければならない。
5	I don’t like to work on a problem unless there is a possibility of getting a clear-cut and unambiguous answer	私は、明確で、曖昧でない答えが得られる可能性がない限り、その問題に取りかかりたいとは思わない。
6	The best part of working on a jigsaw puzzle is putting in that last piece	ジグソーパズルをするうえで一番良い部分は、最後のピースをはめるところだ。
7	A good task is one in which what is to be done and how it is to be done are always clear	良い仕事とは、何が行われるべきか、どのように行われるべきかが常に明確な仕事のことである。

## Discussion

Following the international guideline [20], we developed a Japanese version of the TFA scale and conducted a validation study. Our analysis confirmed that the scale had good structural validity and internal consistency reliability. This developed tool would be useful for educational intervention and the future development of the studies on TFA.

Our analysis showed that both the Japanese and English versions of the TFA scale had a two-factor structure, although the items that made up the factors differed between the two versions. In the Japanese version, unlike the English version, “Q3. I am often uncomfortable with people unless I feel that I can understand their behavior.” was categorized as Factor 1 (desire for certainty), whereas “Q4. Before any important task, I must know how long it will take.” was classified as Factor 2 (willingness to admit discomfort with ambiguity). The contents of Q3 and Q4 were repeatedly reviewed and discussed within the research team, and it was decided that the same factor names as in the English version could be used for Factor 1 and Factor 2. Although it is unclear why the components of each factor differ between the two language versions, there may be due to the nature of cultural differences in handling ambiguity between the USA and Japan [19]. For example, previous studies have suggested that, compared to Western countries, Japan has a “tighter” culture norms with low ambiguity tolerance and is one of the most ambiguity/uncertainty-avoiding cultures [19, 29, 30]. The cultural differences could have an impact on participants’ responses to the questionnaire.

In the present study, the validity and reliability of the scale were demonstrated through a rigorous translation process and validation analysis. The developed inventory could be helpful for future educational interventions and research on TFA. First, at the individual level, the use of the J-TFA scale may promote recognition of the degree of TFA of medical students. Such information will be useful in designing more personalized educational interventions aimed at preventing the decrease in empathy commonly observed in medical students [31, 32], given previous studies indicating a link between TFA and empathy [15, 33]. Additionally, considering that there is an association between TFA and burnout [8], our developed measure may be helpful for identifying individuals with low TFA who are vulnerable to burnout, thus potentially facilitating the prevention of burnout. Second, the scale may be helpful in assessing the impact of learning environment on TFA in medical schools. For example, it could be used to evaluate curricula designed to improve medical students’ TFA and to assess TFA longitudinally over the course of a student’s enrollment.

In future studies, other language versions of the TFA scale should be developed and their psychometric properties should be examined. Research on TFA among medical students in non-English/Japanese-speaking countries would provide deeper insight into TFA. Such findings could lead to an understanding of the impact of culture on TFA.

### Limitations

Our study had potential limitations. First, while this study examined structural validity and internal reliability of consistency, we could not assess other validity (e.g., criterion-related validity) or reliability (e.g., test–retest reliability). Further research should verify these psychometric properties. Second, the values of Cronbach’s alpha for Factor 1 and Factor 2 were relatively small. However, it is not uncommon to observe a low Cronbach’s alpha for scales comprising a limited number of items. This is due to the fact that Cronbach’s alpha is highly sensitive to the number of items included in the scale [34]. Third, distribution of our survey was limited to three medical schools. In future studies, larger numbers of medical schools should be included. Fourth, the use of gift card lottery may introduce bias. However, this appears to be an acceptable method, commonly used in previous studies [35–37]. Fifth, relatively low response rate would raise concerns about selection bias. It is increasingly difficult to achieve high response rates in online surveys [38], with rates often falling to approximately 10% [39]. However, literature indicates that our survey response rates may be sufficient to provide reliable data [40, 41]. Future research should try to maximize the response rates (e.g., generating participant awareness and offering protected time for participants to answer the questionnaire) [42].

## Conclusions

In the present study, in accordance with an international guideline, we translated the original English version of the TFA scale for use in a Japanese undergraduate medical education setting. We examined the structural validity and internal reliability of consistency of the scale through a validation study. Our developed measure may be helpful for future educational interventions and research on TFA.

## Data Availability

The data sets generated and analyzed in this study are available from the corresponding author upon reasonable request.
